# App-based guided problem-solving intervention for adolescent mental health: a pilot cohort study in Indian schools

**DOI:** 10.1136/ebmental-2020-300194

**Published:** 2020-11-18

**Authors:** Pattie P Gonsalves, Eleanor Sara Hodgson, Bhargav Bhat, Rhea Sharma, Abhijeet Jambhale, Daniel Michelson, Vikram Patel

**Affiliations:** 1 Sangath, New Delhi, India; 2 Psychology, University of Sussex, Brighton, Brighton and Hove, UK; 3 Sangath, Porvorim, Goa, India; 4 University of Sussex, Brighton, Brighton and Hove, UK; 5 Harvard Medical School, Boston, Massachusetts, USA

**Keywords:** child & adolescent psychiatry

## Abstract

**Background:**

This paper describes the pilot evaluation of ‘POD Adventures’, a lay counsellor-guided problem-solving intervention delivered via a smartphone app in Indian secondary schools.

**Objective:**

To test the feasibility and acceptability of POD Adventures for adolescents with a felt need for psychological support, and to explore the intervention’s effects on self-reported mental health symptoms, prioritised problems, stress and well-being.

**Methods:**

We used a mixed-methods pre-post cohort design. Participants were self-referred from grades 9–12 in two coeducational government-aided secondary schools in Goa, India. The intervention was delivered in two formats, ‘mixed’ (comprising individual and small group sessions) and ‘group’ (small group sessions only).

**Findings:**

248 participants enrolled in the study and 230 (92.7%) completed the intervention. Outcomes at 4 weeks showed significant improvements on all measures that were maintained at 12 weeks. Large effects were observed on problem severity scores (4 weeks, d=1.47; 12 weeks, d=1.53) while small to moderate effects were seen on mental health symptoms, stress and well-being. 22 students completed qualitative interviews about their experience of the intervention. Participants found POD Adventures easy to use, engaging and helpful in solving their problems. They were satisfied with the guidance provided by the counsellor irrespective of delivery format.

**Conclusions:**

POD Adventures was feasible to deliver with guidance from lay counsellors in Indian schools, acceptable to participants and associated with large improvements in problem severity and mental health symptom severity.

**Clinical implications:**

POD Adventures has promise as an early intervention for adolescents with a felt need for psychological support in low-resource settings.

## Introduction

Mental health problems account for nearly half of the burden of disease in adolescents, with depressive, anxiety and conduct disorders together accounting for over 75% of this burden.^1 2^ The impact of youth mental health problems falls most heavily on low and middle-income countries (LMICs).^3^ India alone contains 20% of the global adolescent population, amounting to some 250 million 10–19 year-olds. At the same time, fewer than 10% of young Indians have access to formal mental health services.^4^ The large mental healthcare gap in India and other LMICs coincides with a rapid boom in telecommunications and internet access.^5^ Young people typically adopt new technologies and use mobile devices and the internet more frequently than older age groups, including for the purpose of accessing health-related information. Digital technologies have therefore been advocated as an important platform for scaling up youth mental healthcare,^6^ with the potential to increase reach, reduce stigma and lower costs compared with conventional clinic-based service models.^7–9^

Notwithstanding varied access and gaps in access or connectivity, especially in rural areas, digital technologies offer unparalleled opportunities for transforming the delivery and use of mental health interventions in low-resource settings.^5^ Recent systematic reviews of mental health apps and game-based approaches for adolescents highlight promising findings for feasibility, acceptability and engagement.^7 9–15^ However, evidence of efficacy is scarce, particularly in low-resource settings.^5 13 16^

This paper describes the pilot evaluation of ‘POD Adventures’, a problem-solving app intervention delivered in Indian secondary schools with guidance provided by lay counsellors. This approach integrates face-to-face contact with self-guided digital content and is consistent with findings that human facilitation can optimise engagement with and outcomes of digital interventions.^10 17^ POD Adventures is part of the PRIDE research programme (2016–2021) that has been developing and evaluating transdiagnostic psychological interventions for common adolescent mental health problems in India.^18^ Previous PRIDE studies^19 20^ revealed a high demand for psychological support among secondary school pupils, the majority of whom did not meet conventional clinical thresholds for mental disorders. Keeping this wider group in mind, POD Adventures was conceptualised as an open-access, early intervention to promote adaptive coping and to mitigate risks for developing more severe and socially disabling mental health problems in the longer term. The app was collaboratively designed with adolescents through an iterative and person-centred approach, incorporating insights from a range of user consultations across a period of 18 months prior to conducting this study.^21^

The aim of the current study was to test the feasibility, acceptability and potential effects of POD Adventures as a guided app-based intervention for adolescents with a felt need for psychological support irrespective of assessed clinical severity. Our ultimate goal was to refine the intervention based on these findings in preparation for a rigorous evaluation through a randomised controlled trial.

## Methods

### Setting

The study was conducted in two coeducational, government-aided, English-medium secondary schools in Goa, India, without established counselling services. Goa is one of India’s most highly urbanised states and offered a relevant context in which to evaluate a technology-enabled intervention intended for low-resource settings. The schools comprised adolescents from both centrally located urban and remote rural areas of the state.

### Design

A pre-post cohort study used an iterative mixed-methods design with concurrent data collection and analysis aligned with guidance for complex evaluations.^22^ This enabled rapid feedback of emergent findings and concurrent refinements to the intervention and its delivery. A secondary objective was to assess indicative outcomes at 4 and 12 weeks after entry into the study.

### Participants

#### Eligibility

Eligible participants were students in grades 9–12 (aged 13–19 years) who self-referred for psychological help with perceived stress, and were proficient in written and spoken English or Konkani (as needed to participate fully in study procedures and the intervention). Participants were excluded if there was an elevated risk of self-harm or suicide requiring external referral. Risk was identified using a brief screening questionnaire followed by a structured interview-based assessment where indicated.

#### Recruitment

The sampling frame consisted of all students from the eligible classes in the two participating schools. Recruitment involved a brief 20–30 min sensitisation session, delivered to individual classes, which promoted the intervention as a ‘stress reduction and problem-solving program’. These one-off sessions were conducted by a counsellor using a digital slideshow to support discussion about commonly experienced stressful problems and to provide a brief description of the intervention and referral process. Students were invited to self-refer by any one of three methods: (1) completing and returning a referral form at the classroom session; (2) using a ‘drop-box’ for referral forms at other times; or (3) approaching a member of the study team during school hours. Recruitment remained open from July 2019 until January 2020.

### Intervention

#### Content

The intervention comprised a smartphone app, ‘POD Adventures’, guided by a lay counsellor. The app was built around three problem-solving steps that had been developed and evaluated for use in non-digital intervention formats through the PRIDE programme^19 20^: (1) ‘Problem identification’; (2) ‘Option generation’; and (3) creating a ‘Do it’ plan. The app content was organised in two sections: (1) *Adventures*, in which problem-solving concepts and methods were taught through gamified stories along with guided practice of emotion regulation strategies; and (2) *My POD,* in which a series of questions scaffolded the participant through step-by-step problem solving for their own problems (table 1).

**Table 1:** Intervention structure, content and delivery

	App content		Delivery and guidance	
			Mixed format	Group format
Session 1	‘P’ step of problem identification and prioritisation; practice of emotion regulation exercise of ‘colour breathing’, a guided breathing exercise with visualisation.	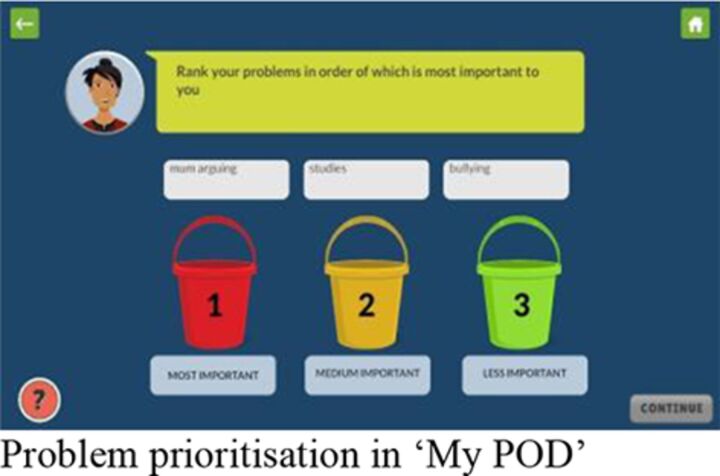	**Individual** 1:1 onboarding to orient the student to the app and build rapport. Independent gameplay of the app with support and troubleshooting as required.	**Group** Group onboarding to orient students to the app and build rapport. Independent gameplay of the app with support and troubleshooting as required.
Session 2	‘O’ step of option generation, weighing pros and cons and selecting the best option; practice of emotion regulation exercise of mindful stretching.	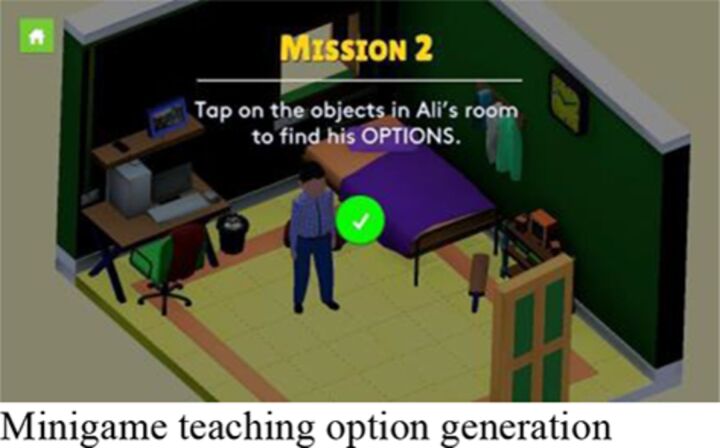	**Group** Independent gameplay of the app with support and troubleshooting as required.	**Group** Independent gameplay of the app with support and troubleshooting as required.
Session 3	‘D’ step of making a ‘do it’ plan for selected option; practice of emotion regulation exercise of ‘happy place’ guided imagery exercise of imagining a place they feel happy, safe and calm.	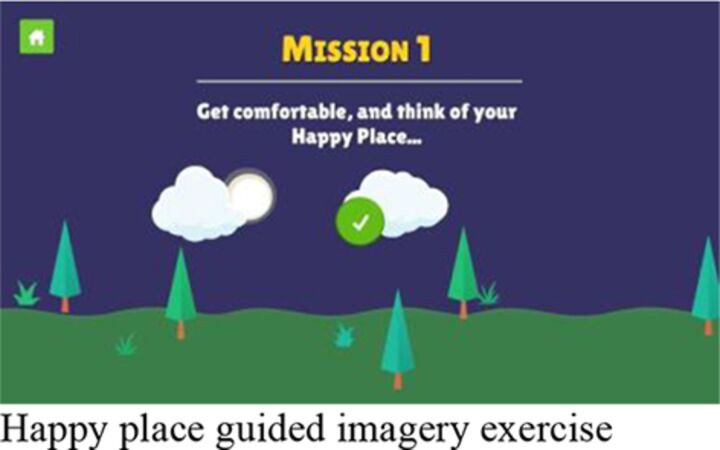	**Group** Independent gameplay of the app with support and troubleshooting as required.	**Group** Independent gameplay of the app with support and troubleshooting as required.
Session 4	Review the outcome of the ‘do it’ plan and make a revised plan where necessary; practice of emotion regulation exercise of the participant’s choice.	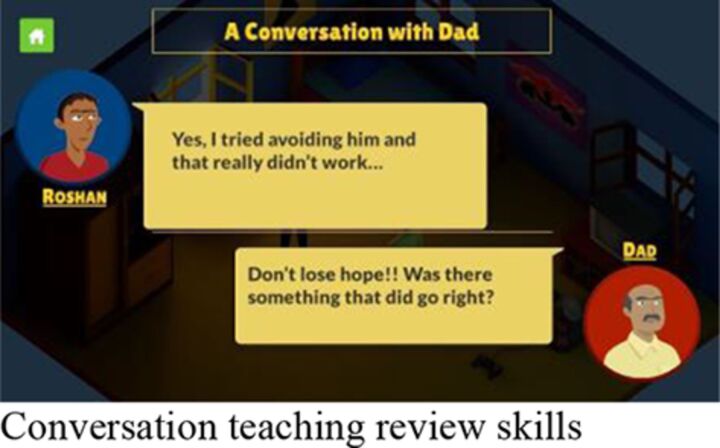	**Individual** 1:1 review of student’s progress and understanding of POD steps. Independent gameplay of the app with support and troubleshooting as required.	**Group** Group review of students’ progress and understanding of POD steps. Independent gameplay of the app with support and troubleshooting as required.

#### Delivery

POD Adventures was delivered during school hours in school-allotted rooms and on devices provided by the study team. Participants could not access the app outside these sessions. The app was designed to function offline (ie, no internet connectivity required when in use) due to the lack of reliable internet connectivity in Goa. The app was offered in English with Konkani (local language) voice-over. The intervention delivery schedule consisted of four 30–40 min sessions over 2–3 weeks. The first session included an ‘onboarding’ that provided an overview of the programme and introduction to the app (table 1). The fourth session concluded with a ‘review’ of students’ progress to consolidate learning. The onboarding and review lasted approximately 10 min each and were implemented by counsellors according to detailed scripts. For the remainder of the sessions, participants used the app independently with counsellor guidance provided as and when required. Guidance entailed explanation and troubleshooting of any app content or technical issues.

To understand the efficiency of delivery (as part of feasibility assessment), the intervention was delivered through two delivery formats (table 1), the order of which was randomly chosen. In the first instance, a ‘mixed’ delivery format was offered to all eligible study participants over a period of 2 months. This format comprised individual sessions at the beginning and end (sessions 1 and 4), while sessions 2 and 3 were conducted in groups of up to six participants in which participants used the app independently while a counsellor was available for support as required. For the remaining 5-month period of the study, we used a ‘group’ format for all sessions. Scripts for the onboarding and review were adapted to each delivery format but covered the same overall content.

#### Counsellors

Guidance was provided by four bilingual English and Konkani-speaking lay counsellors. They were college graduates with 2 years of experience in delivering a face-to-face variant of the same problem-solving intervention^19^ but were not formally qualified to provide support for problems outside the scope of what this intervention offered. Counsellors received a 4-day office-based training built around a printed intervention manual. Supervision consisted of weekly peer group supervision meetings (lasting approximately 1 hour), moderated by a clinical psychologist. In each meeting, counsellors rated and discussed selected audio-recorded sessions using a session fidelity scale.

### Measures and collection procedures

Feasibility of research procedures was assessed through routinely logged rates of referrals, eligibility, assent and consent, and completion of outcome assessments. Feasibility of the intervention was assessed using data on session attendance, session duration and intervention completion (ie, attendance at all four sessions) and fidelity ratings of onboarding and review discussions.

User satisfaction was assessed using the Client Satisfaction Questionnaire-8 (CSQ-8)^23^ and four additional questions about the app ((1) The game has increased my ability to cope with problems on my own; (2) The game was easy to use; (3) The information in the game was easy to understand; (4) The game was fun and interesting to use) which obtained user ratings of helpfulness, usability, ease of understanding and enjoyment. Semistructured qualitative interviews were conducted by three study authors (PPG, RS and AJ) with a purposively selected sample of students (n=22) who completed the intervention. We aimed to approximate equal quotas with regard to age, gender and delivery format. Questions covered acceptability of attending school-based sessions, using the allocated smartphone, preferences for specific app features, game usability, helpfulness of the app content and experiences of counsellor guidance. Interviews were conducted by two study authors (PPG and RS) and audio recorded, transcribed verbatim and translated to English where necessary.

Clinical outcomes were assessed using four validated self-report questionnaires that measured psychosocial problem severity (Youth Top Problems (YTP)),^24^ mental health symptoms (Strengths and Difficulties Questionnaire (SDQ)),^25^ perceived stress (Perceived Stress Scale (PSS))^26^ and well-being (Short Warwick-Edinburgh Mental Well-Being Scale (SWEMWBS)).^27^ All measures have been used in previous PRIDE studies.^19 20^ Outcomes were collected at baseline, 4 and 12 weeks after baseline.

### Analysis

Quantitative process indicators were described using frequencies, means, SDs and proportions. Analysis of clinical outcome measures involved comparisons of pre-post scores using paired t-tests and was restricted to participants who completed baseline and endpoint assessments. Outcome measures were compared between baseline and the 4 and 12-week endpoints. Subgroup analyses were conducted by baseline SDQ severity to examine the impact of the intervention separately for those in the subthreshold and case severity ranges using Indian SDQ cut-offs of 19 for girls and 20 for boys.^28^ Subgroup analyses were also conducted for each delivery format (mixed and group).

Student interviews were analysed using an integrated inductive-deductive approach to thematic analysis.^29^ Familiarisation with the data set was followed by line-by-line coding of a subset of manuscripts by two coauthors (PPG and RS). This created an initial list of categories and codes. An iterative process of coding additional manuscripts, reviewing and revising the coding framework was followed before arriving at a final set of themes, categories and codes. Interview transcripts were analysed using NVivo V.12.

## Results

### Uptake

**Figure 1:**
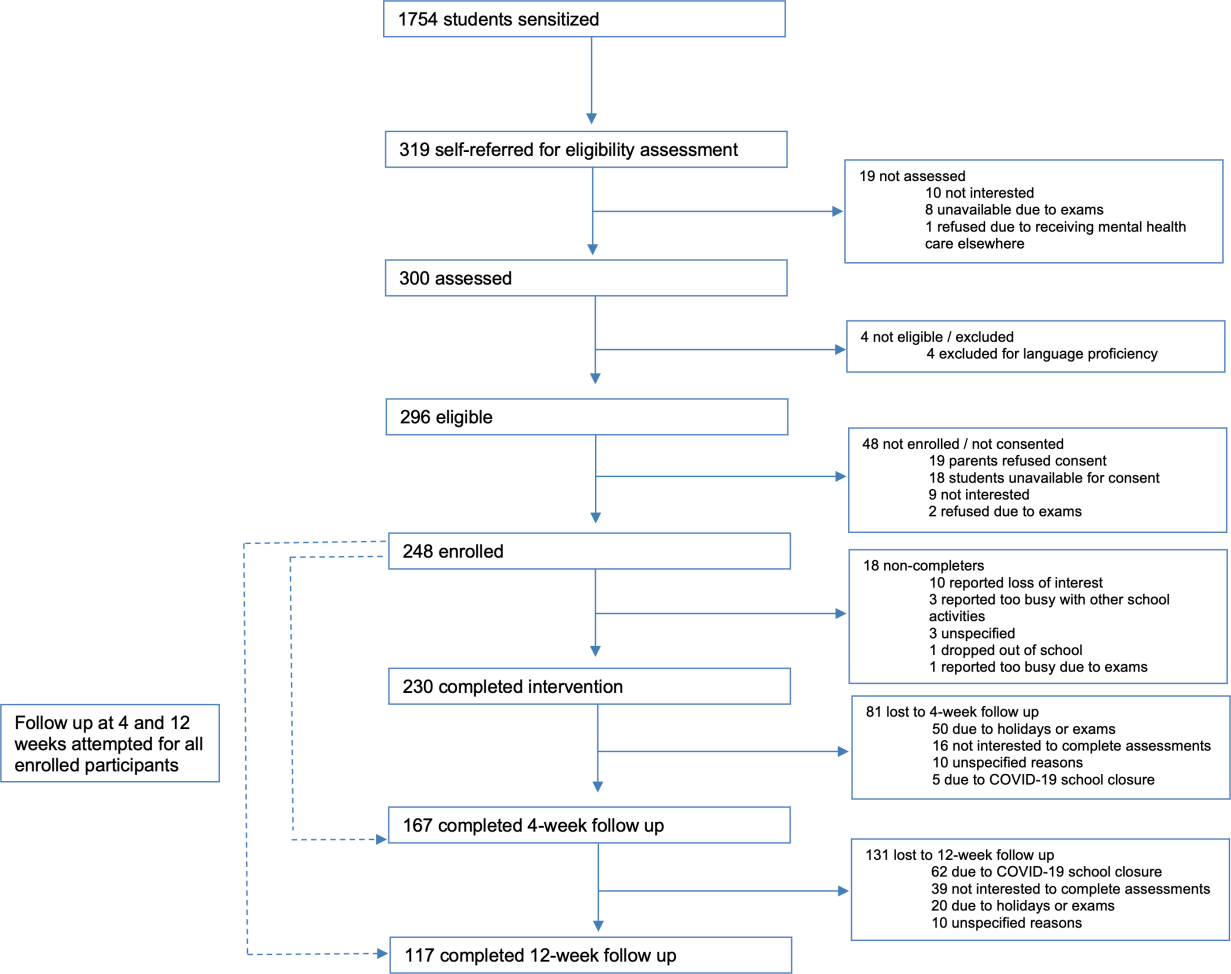
Participant flow.

Sensitisation sessions were conducted in 36 classrooms with 1754 students, from which n=319 (18.2%) referrals were generated (all self-initiated by students). Most of these self-referrals were made at the end of classroom sessions (n=207); the remainder occurred when a student approached a researcher (n=61) or deposited a referral form in a drop box (n=51). Rates of referral differed by grade, with very few participants from grade 10 (n=8, 2.6%) and grade 12 (n=34, 10.7%), which are national examination grades. From the referred sample, n=4 students (1.4%) were excluded on the basis of literacy, no students were excluded due to risk and n=248 (83.7%) provided assent and parental consent to participate in the study. Reasons for non-consent were mostly due to loss to follow-up, lack of interest or examinations (n=29) followed by parents declining consent (n=19) (figure 1).

### Demographics

Consenting participants were aged 13–19 years (mean 15.57 years; 124 male and 124 female). The SDQ Total Difficulties mean score at baseline for the entire sample (n=248) was 15.90, SD=5.66. One hundred and seventy-seven participants (71.4%) scored in the subthreshold range (SDQ Total Difficulties mean=13.13, SD=3.77) while the remainder of the sample, n=71 (28.6%), were at or above the case severity cut-off score (SDQ Total Difficulties mean=22.79, SD=3.17). One hundred and fifty-seven participants (63.3%) reported clinically significant functional impairment on the SDQ Impact Supplement (SDQ Impact mean=3.16, SD=2.02).

### Completion

Two hundred and thirty participants (92.7%) completed four sessions within the designated time frame of 2–3 weeks. Non-completers tended to be slightly older (completer mean age=15.51 years; non-completer mean age=16.33 years; p=0.009), female (female non-completers, n=13 (10.5%); male non-completers, n=5 (4.0%); p=0.05) and reported higher SDQ Total Difficulties scores at baseline (completer mean score=15.66, SD=5.46; non-completer mean score=18.83, SD=7.37; p=0.02). Reasons for non-completion were loss of interest (n=10), examinations (n=1), other school activities (n=3), dropped out from school (n=1) and unknown (n=3).

### Delivery

Most participants (n=173, 69.7%) received the group delivery format and n=57 (22.9%) took part in the mixed format. The group format was associated with less cumulative session time over the course of the intervention (group, mean=1 hour 36 min; mixed n=57, mean=2 hours 15 min) and less counsellor time per student (group, mean=9.46 min; mixed, mean=24.49 min). The mean number of participants who attended group sessions was 3 instead of the planned 5–6 students, largely due to classroom scheduling challenges. Fidelity ratings of 71 sessions revealed a high level of faithfulness to the scripts for the onboarding and review (mean 1.77; SD 0.20; inter-rater correlation of 0.67).

### Satisfaction

Service satisfaction scores ranged from good to excellent on the CSQ-8 (CSQ-8: M=26.20; SD=3.20; range=18–32) and the four app-specific questions. The item-wise mean for the CSQ-8 was 3.28, SD=0.40 (maximum of 4) and the app-specific mean score was 3.66, SD=0.34 (maximum of 4). Nearly all respondents (n=157; 91.3%) felt that the programme and app had helped them to deal more effectively with their problems. There was a trend towards higher satisfaction ratings among participants who received the mixed format (group, mean CSQ=25.89 (SD=3.37); mixed, mean CSQ=26.96 (SD=2.60); p=0.05). Analysis of satisfaction by age and gender revealed no significant associations.

### Clinical outcomes

Follow-up assessments at 4 weeks were completed for n=167 (67.3%) of the sample. Missing assessments were due to timing constraints around examinations or holidays (n=50), participants who did not want to complete assessments (n=16), early school closure due to COVID-19 (n=5) and non-attendance for unspecified reasons (n=10). Follow-up assessments at 12 weeks were completed for n=117 (47.2%) of the sample. Missed assessments were due to early school closure due to COVID-19 (n=62), participants who did not want to complete assessments (n=39), holidays or examinations (n=20) and unspecified reasons (n=10). No significant differences were found for baseline characteristics of age, gender, problem severity or mental health symptoms for those who did and did not complete end line or follow-up assessments.

Compared with baseline, outcomes at 4 weeks showed significant improvements on all measures (table 2). These improvements were maintained at 12 weeks. Large effects were observed on YTP scores at both endpoints relative to baseline (4 weeks, d=1.47; 12 weeks, d=1.53) while small to moderate effects were seen on the SDQ Total Difficulties, PSS-4 and SWEMWBS. Similar improvements were observed across outcomes for both delivery formats: mixed, YTP at 4 weeks d=1.3, 95% CI 0.91 to 1.67; YTP at 12 weeks d=1.36, 95% CI 0.93 to 1.76; SDQ Total Difficulties at 4 weeks d=0.50, 95% CI 0.20 to 0.79; SDQ Total Difficulties at 12 weeks d=0.64, 95% CI 0.31 to 0.96; and group, YTP at 4 weeks d=1.56, 95% CI 1.29 to 1.83; YTP at 12 weeks d=1.64, 95% CI 1.28 to 1.98; SDQ Total Difficulties at 4 weeks d=0.56, 95% CI 0.36 to 0.75; SDQ Total Difficulties at 12 weeks d=0.67, 95% CI 0.21 to 0.69. There were no significant differences in outcomes on the basis of age or gender.

**Table 2:** Clinical outcomes

		YTP	SDQ	PSS-4	SWEMWBS
4-week pre-post (n=167)	Baseline Mean (SD)	6.63 (2.07)	15.71 (5.56)	7.62 (2.66)	22.21 (4.35)
	4 weeks Mean (SD)	3.27 (1.72)	13.23 (5.40)	6.32 (2.47)	23.80 (4.48)
	Mean difference 95% CI	3.36 (3.01 to 3.71)	2.48 (1.78 to 3.17)	1.30 (0.82 to 1.78)	−1.59 (−2.37 to −0.80)
	P value	<0.001	<0.001	<0.001	<0.001
	Effect size (95% CI)	d=1.47 (1.25 to 1.69)	d=0.54 (0.38 to 0.70)	d=0.41 (0.25 to 0.56)	d=0.31 (0.15 to 0.46)
12-week pre-post (n=117)	Baseline Mean (SD)	6.62 (2.08)	15.93 (5.42)	7.66 (2.41)	21.74 (4.06)
	12 weeks Mean (SD)	2.93 (1.65)	12.60 (5.65)	6.19 (2.52)	23.23 (5.02)
	Mean difference 95% CI	3.70 (3.25 to 4.14)	3.33 (2.41 to 4.26)	1.47 (0.90 to 2.04)	−1.49 (−2.42 to −0.56)
	P value	<0.001	<0.000	<0.001	<0.002
	Effect size (95% CI)	d=1.53 (1.26 to 1.79)	d=0.66 (0.45 to 0.85)	d=0.47 (0.28 to 0.66)	d=0.29 (0.10 to 0.47)

PSS, Perceived Stress Scale; SDQ, Strengths and Difficulties Questionnaire; SWEMWBS, Short Warwick-Edinburgh Mental Well-Being Scale; YTP, Youth Top Problems.

Subgroup analyses by SDQ Total Difficulties baseline score (subthreshold or case severity range) showed statistically significant improvements at 4 weeks for both groups on the SDQ Total Difficulties scale (subthreshold (n=122) baseline=13.15, 4 weeks=11.65, t=3.99, p<0.001; case severity (n=45) baseline=22.67, 4 weeks=17.54, t=7.50, p<0.001) and YTP (subthreshold (n=122) baseline=6.28, 4 weeks=3.14, t=14.69, p<0.001; case severity (n=45) baseline=7.64, 4 weeks=3.60, t=12.88, p<0.001).

### Intervention experience: participant interviews

Exit interviews were conducted with 22 students who completed the intervention (aged 13–17 years; 11 male, 11 female). Three themes emerged from the interviews: (1) experiences of digital content; (2) interactions with peers and counsellors; and (3) impacts (table 3).

**Table 3:** Illustrative quotes from exit interviews with participants

**Experiences of digital content**	
Look and feel	Engagement *It was more visually attractive and better when it’s visual rather than the normal books and all*. Novelty *I would say it was quite helpful, it was innovative, like something new, something different*. Privacy *The game has a nice quality, it keeps information private with headphones. This helps in maintaining privacy*.
Valued features	Learning through relatable stories *After seeing the story I came to know what was happening with me too, that it was the same thing, almost the same situation that was happening related to bullying and all. I had the same problem, so I figured out how to get help and so everything has become good now*. Relaxation and emotion regulation *Breathing out and breathing in. That is the thing I learnt a lot. That’s what made my problem go down a bit. My friends would ask me what are you doing, ‘you just kept quiet suddenly’ but I am practicing it*.
Usability	Game mechanics and typing difficulties *I had no real difficulties, but sometimes while putting in passwords it was difficult and the teacher would handle it*. Easy to use *Initially I was thinking that when we use the mobile to play the game it will be difficult but when I used it, it was easy*.
Improvements	Improvements to Adventures *More stories… stories feel like how lessons are. If the lesson is good so then we go to the end of the lesson, but if the lesson is boring then we don’t, so those are the kinds of stories, like the stories were interesting*. Improvements to My- POD *The same question was asked but in two different ways. I had to give the same answers, so I didn’t really understand why, I had to write it twice*.
**Interactions with peers and counsellors**	
Social acceptability	Influence of other students joining the programme *We all signed up together and it was good… We even recommended it to people from other schools but they were not from your group…* Leaving class for mental health support *Initially when I was called from class to go to a session my friends used to ask me where I am going. I am not ashamed to tell them that I am going to the counsellor. It’s OK, everyone needs help! Maybe it’s just in a different way. The first time I just kept my mouth shut and didn’t say, but when I went down for interval my friends asked me again, and I didn’t want to avoid them or make it look like I was hiding stuff. I just said that I was with the counsellor. I thought I would get a very bad reaction but they actually asked me what happened! They were concerned about me*.
		Guidance and delivery	Comparisons with face-to-face counselling *The game was really good, better than talking to someone*. *Sometimes you don’t like sharing or talking to someone*. *In the game you are by yourself, you can put in what you want. You don’t have to worry about another person*. *The app helps with its stress relieving methods but what comes from the person is much better than that. I wouldn’t suggest it as much as talking to the person. That’s much better, it makes you feel better. At least in my case it made me feel much better*. Preference for individual or group sessions *It felt good in the group. When I am individually doing it I get bored because nobody is there, if there is some movement of others then it feels a little good*. Counsellor guidance valued *It was very encouraging. It was very helpful to have someone encouraging you to go through with your solutions*. *Counsellors would also help in the middle if we needed. They were like friends and telling them our problems or problem solving wasn’t scary. They would do it nicely*.
**Impacts**			
Direct problem resolution	*The problems I wrote are not affecting me in my life anymore, so now if I get a problem I think of myself as a POD master! I realised that there are options in life and we have gone through that…to test one of them and which is the best so we can just try out them*.
Ongoing practice	*Now I am doing regular practice sometimes because of which I feel relaxed and also getting less distraction so now I can give attention*.
Increase in knowledge and skills	*The top most thing I learned was about the time table, and to make my own*.
Future use of learning	*When I myself or any friends or even if my family has any problem then I can tell them this idea*.

#### Digital content

Participants highlighted the overall novelty of using a smartphone to learn skills that they could apply in their everyday lives. The level of privacy and ability to work independently were also mentioned favourably. Learning through characters’ stories was a highly valued feature and a larger bank of stories was noted by several participants as a potential improvement. The app was generally considered easy to use but a few participants identified certain confusing game mechanics (eg, ‘drag and drop’) and issues related to typing and difficult login passwords.

#### Interactions

Participation in the intervention was found to be socially acceptable with participants noting the positive influence of seeing other students signing up. Participants denied any difficulties related to teasing or stigma while leaving class to join sessions. Participants felt adequately supported by the counsellor’s input, and commented positively on counsellor qualities such as helpfulness and friendliness. Those participants who experienced the mixed delivery format did not show a clear preference for either the group or individual session options. Moreover, those who received only group sessions were pleased with the level of guidance available and did not believe that an individual session option would have been preferable. One participant expressed that the guidance from the counsellor was more helpful in making them feel better than the content of the app.

#### Impacts

Nearly all participants felt that the programme had positively impacted their prioritised problem. Some participants specifically mentioned more generalised benefits result from enhanced coping skills (eg, time management and techniques for emotion regulation) and indicated ongoing practice of these skills.

## Discussion

This study assessed the feasibility, acceptability and indicative outcomes of an app-based guided problem-solving intervention for adolescents ahead of a future trial. Overall, participants expressed a high degree of satisfaction with the experience of using the app and with the associated guidance provided by the supporting counsellors. Moreover, nearly one in every five students who attended classroom sensitisation sessions about the app decided to self-refer, suggesting a high degree of social acceptability that was corroborated by participant interviews. The high demand for the intervention is an important finding given the evidence that stigma can serve as a major barrier to uptake of school-based mental health interventions.^30^ It is notable that adolescents highlighted privacy as one of the preferred features of the app and this aspect was also emphasised as part of the sensitisation sessions. Even though the intervention was delivered in a group format for at least some of the sessions, adolescents were not required to disclose personal information to peers or directly to counsellors. More generally, our findings contribute to the evidence that digital formats may be appealing to adolescents regardless of exposure and access to technology, although most of the previous research in this area originates from high-income countries.^7 11 12^

The intervention completion rate (92%) is noteworthy as low levels of adherence are frequently reported in digital adolescent mental health interventions, including in school settings.^7 9 14 15^ In line with our observations, the literature suggests that interventions involving regular interactions with a therapist or that are completed in a supervised setting tend to be more acceptable and effective and have higher rates of engagement and completion.^9 10^ The use of a person-centred approach in the app’s development, entailing adolescent codesign activities and extensive user testing, may also have enhanced overall acceptability.^21^ Although rates of non-completion were low, it is notable that female and older participants with higher SDQ scores were more likely to discontinue. There were no significant differences in satisfaction according to age or gender suggesting that this might not reflect broader trends in acceptability and it will be important to monitor these differential effects in future evaluations.

Participants valued guidance from the counsellors, whether it was provided individually or in small groups. Though satisfaction scores showed a trend in favour of the mixed delivery format, there were also indications that the group format provided a helpful normalising context in which participants drew encouragement from seeing peers engaged in the programme. Further, both formats of the intervention produced similar improvements across outcome measures. Given that group delivery was demonstrably more efficient than the mixed format (requiring less counsellor guidance time per individual student), there is a compelling argument for the group format being the default option for future studies.

POD Adventures showed potential for clinical impact across several outcome domains. Problem severity, mental health symptoms, stress and well-being were significantly improved at 4 weeks and these gains maintained at 12-week follow-up. Interviews corroborated these improvements, particularly with respect to resolution of focal problems. Participants also spoke about ongoing practice and recognised opportunities for future use of newly learnt skills, suggesting that the intervention may help build resilience to future problems in spite of its brief delivery schedule. We also note that similar effects on problem severity and mental health symptoms were observed irrespective of participants’ baseline symptom severity. This is particularly relevant in India where previous PRIDE studies have demonstrated high demand for counselling from students who do not meet clinical thresholds^18–20^ and where early interventions for emerging mental health problems are scarcely available.^4^

In deciding on potential intervention modifications, we considered findings from this study as well as overall programme aim and resources required to make the changes. The resulting changes consisted of: (1) *content changes:* revisions to text and story components to enhance acceptability; (2) *user interface and gamification changes:* updating of the colour scheme to make it more visually appealing and adding additional instruction screens; and (3) ‘*back end’ technical changes:* use of simpler passwords and building online functionality allowing for real-time data syncing and remote delivery of the intervention. Finally, group delivery was selected as the optimal modality for a future evaluation.

### Strengths and limitations

The study did not use a control group so we cannot rule out the possibility of regression to the mean or improved outcomes due to factors other than the specific effects of the intervention. In addition, outcomes were assessed via self-report and so it is possible that participants may have reported more favourable responses because they were in regular contact with the counsellors and researchers. Follow-up at both endpoints was impacted by participants who did not want to complete assessments and poor follow-up at 12 weeks due to early school closures which may have biased our results and further limits conclusions about potential impacts which should be interpreted with caution. We will therefore consider briefer and more engaging methods to collect participant outcomes and follow them up for the future study. On the other hand, and in line with guidance for formative evaluations of complex interventions,^21^ we supplemented quantitative data with in-depth qualitative interviews to generate rich descriptions of intervention acceptability, feasibility and impacts.

## Conclusions

This mixed-methods evaluation found that POD Adventures was feasible, acceptable and potentially effective at improving mental health symptoms and associated outcomes among help-seeking adolescents in a low-resource context. POD Adventures might be especially helpful when adolescents first begin to experience distress. The app can be delivered efficiently in schools—and possibly other settings—using a low-intensity group format that requires minimal guidance. Especially in light of COVID-19, exploring remote digital delivery of such an intervention either independently or with remote guidance may be of particular relevance.
